# Genome-Wide Identification of Binding Motifs and Drought-Responsive Target Genes by the Transcription Factor ZmNAC20 in Maize

**DOI:** 10.3390/plants15030423

**Published:** 2026-01-30

**Authors:** Tengao Xu, Yuan Zhang, Yizhuo Zhang, Qi Huang, Miao Wang, Jiahao Yang, Hui Zhang, Wei Wang, Hui Liu

**Affiliations:** State Key Laboratory of High-Efficiency Production of Wheat-Maize Double Cropping, College of Life Sciences, Henan Agricultural University, Zhengzhou 450046, China

**Keywords:** ZmNAC20, drought stress, DAP-seq, RNA-seq, transcription factor, maize

## Abstract

Drought stress severely limits maize growth, and enhancing drought resistance remains a central objective of crop improvement. Plant-specific NAC (NAM/ATAF/CUC) transcription factors are critical regulators of abiotic stress responses. Previously, we identified ZmNAC20—a gene rapidly induced by both drought and abscisic acid (ABA)—as a positive regulator of stomatal closure and drought survival when over-expressed; however, its direct target genes and downstream regulatory network remained elusive. Here, we used DAP-seq to identify 3537 ZmNAC20 binding peaks genome-wide, revealing four enriched cis-motifs and 1439 target genes. GO and KEGG analyses showed pronounced enrichment in intracellular signal transduction. The intersection of RNA-seq of drought-stressed ZmNAC20-over-expressing and wild-type B104 seedlings with DAP-seq outputs defined 80 potential direct targets (48 up-, 32 down-regulated). Among the activated genes, ZmNAC20 binds and up-regulates genes of the ROS-producing enzyme *ZmRBOH8*, BURP domain-containing protein *ZmBURP4*, gibberellin (GA) catabolic enzyme *ZmGA2OX6*, and HD-Zip transcription factor *ZmHB56*. These results unveil a multi-layered drought-responsive network through which ZmNAC20 integrates hormone and ROS signaling, providing a molecular blueprint for breeding drought-resilient maize.

## 1. Introduction

Maize (*Zea mays* L.) is one of the most important crops worldwide, and both its yield and quality are strongly influenced by environmental stresses [1]. With global climate change, the frequency and intensity of drought events are increasing [2], posing a serious threat to stable maize production [3]. Drought not only restricts water and nutrient uptake but also inhibits photosynthesis, promotes the accumulation of reactive oxygen species (ROS), and disrupts normal metabolism [4,5]. Ultimately, these effects lead to arrested growth, impaired pollination, and significant yield losses [3]. To cope with drought stress, plants have evolved a sophisticated and finely tuned molecular regulatory network during their long history of adaptation [6]. Within this network, transcription factors play a central role in stress sensing and downstream signaling by modulating the expression of drought-responsive genes [7]. Among the various transcription factor families, the NAC (NAM, ATAF, and CUC) family has attracted particular attention because of its pleiotropic functions in stress responses (drought, cold, heat, and salt) [8,9,10,11], and development checkpoints (seed iron loading, leaf senescence, and fruit ripening) [12,13,14,15,16], indicating that NAC factors serve as central integrators of environmental cues and developmental programs, thereby offering high-value targets for engineering stress-resilient, nutrient-enriched, and shelf life-extended crops. NAC transcription factors belong to a large, plant-specific family of regulatory proteins whose name reflects the conserved domain first identified in Petunia NAM (no apical meristem), Arabidopsis ATAF1/ATAF2, and CUC (cup-shaped cotyledon) genes [17]. A typical NAC protein possesses a highly conserved N-terminal NAC domain of ~150–160 amino acids that can be subdivided into five subdomains (A–E) and is primarily responsible for DNA binding, dimerization, and signal transduction. By contrast, the C-terminal region is highly divergent and usually functions as a transcriptional activator or repressor that confers regulatory specificity [17]. Based on sequence divergence within the NAC domain and phylogenetic relationships, the family can be classified into multiple subgroups whose members exhibit distinct functions in plant growth, development, and stress responses [18].

A growing number of recent studies show that NAC transcription factors can significantly enhance drought tolerance in wheat [19], maize [8], rice [20], soybean [21], tobacco [22], and cotton [23], suggesting a conserved and potent regulatory role of NAC proteins in orchestrating drought stress adaptation across diverse plant species. Reports show that NAC transcription factors enhance plant water retention capacity and stress resistance by modulating the biosynthesis of osmolytes such as proline and soluble sugars, augmenting antioxidant enzyme activities, and regulating ABA-dependent signaling pathways [24,25,26,27]. In addition, some NAC genes have been shown to interact with other transcription factors, forming complex regulatory networks that fine-tune the expression of downstream stress-responsive genes [20,28]. A subset of NAC transcription factors carry a conserved NAC domain at the N-terminus and a transmembrane anchor at the C-terminus. Upon environmental stimuli, these membrane-tethered factors are released and rapidly imported into the nucleus, where they activate stress-responsive genes [29]. These findings not only illuminate the multifaceted functions of NAC genes in drought stress responses but also provide an important reference and theoretical foundation for elucidating the molecular mechanisms underlying the drought resistance of NAC transcription factors.

In maize, genome-wide surveys have identified more than 116 NAC transcription factor members [30], and some of them have been confirmed to respond to drought stress [31]. Among these drought-responsive *NAC* genes, several have now been functionally validated. Over-expression of *ZmNAC111* significantly enhances drought tolerance in maize, and natural variation in the *ZmNAC111* promoter is closely associated with drought resistance [32]. *ZmNAC33* is up-regulated by drought stress and ABA treatment; its over-expression in Arabidopsis improves drought tolerance [33]. *ZmNAC55* is induced by drought, salt, cold stresses and ABA. Over-expressing *ZmNAC55* in *Arabidopsis* markedly increases drought resistance [34]. *ZmNAC49* is rapidly up-regulated by drought stress. Over-expression of *ZmNAC49* in maize reduces both stomatal conductance and stomatal density. Further analysis revealed that ZmNAC49 directly binds to the promoter of *ZmMUTE* and represses its transcription, thereby decreasing stomatal density. Thus, ZmNAC49 enhances drought tolerance mainly by modulating stomatal density [35]. Over-expression of the NAC transcription factor *ZmNST3* significantly improves drought tolerance in maize. ChIP-seq assays showed that ZmNST3 directly associates with the promoters of *CESA5* and *CESA2A* and activates their expression, leading to elevated transcript levels of genes involved in secondary cell-wall cellulose biosynthesis [36]. The NAC transcription factor *ZmNUT1* is predominantly expressed in the xylem of roots, stems and leaves. Loss of *ZmNUT1* disrupts water transport. ZmNUT1 regulates xylem development and consequently water transport by directly controlling cellulose synthase genes and cysteine protease genes [37]. Natural variation in the non-coding region of *ZmNAC080308* is associated with drought tolerance, and over-expression of *ZmNAC080308* in Arabidopsis markedly enhances drought resistance [38]. ZmSNAC13 enhances drought tolerance by promoting the expression of *PYL9* and *DREB3* [39]. ZmNAC84 improves drought resistance through direct regulation of *ZmSOD2* [40].

Because NAC transcription factors are central regulators of drought tolerance, elucidating the complete ZmNAC-centered regulatory network is indispensable for advancing our understanding of drought resistance biology and for guiding molecular breeding aimed at developing drought-resilient maize cultivars. DAP-seq (DNA affinity purification sequencing) and RNA-seq (RNA sequencing) are two cornerstone high-throughput technologies for dissecting transcription factor regulatory networks, and they offer complementary advantages in functional genomics [11]. DAP-seq employs an in vitro-synthesized target transcription factor to capture genomic DNA fragments, followed by high-throughput sequencing to map transcription factor binding sites genome-wide [11]. Integrating DAP-seq with RNA-seq provides dual validation of direct transcription factor binding and transcriptional responses: DAP-seq-defined binding sites represent candidate regulatory targets, whereas RNA-seq confirms how these targets change in expression upon transcription factor activation or repression [16,41,42]. This combined strategy not only builds transcription factor–target gene regulatory networks, but also, when coupled with gene functional annotation and pathway analysis, elucidates the mechanisms by which transcription factors govern complex biological processes such as stress responses and developmental regulation. Previously, we showed that *ZmNAC20* is rapidly up-regulated by drought stress and ABA, and that its over-expression enhances maize drought tolerance by promoting stomatal closure; nevertheless, its direct target genes and the downstream regulatory network remain unknown. Here, the study aims to integrate DAP-seq and RNA-seq to construct a genome-wide ZmNAC20-centered regulatory network that underpins drought resistance in maize.

## 2. Results

### 2.1. Genome-Wide Occupancy Landscape of ZmNAC20 Revealed by DAP-Seq

In our previous study, ZmNAC20 was identified as a positive regulator of drought resistance in maize [26]. To dissect the genome-wide transcriptional circuitry governed by ZmNAC20, we employed DAP-seq to map its native binding landscape. DAP-seq has recently emerged as a robust tool for capturing genome-wide DNA binding sites of transcription factors in the native chromatin context [16,41,42]. For DAP-seq analysis, the recombinant ZmNAC20 fused to the HaloTag sequence was used to retrieve ZmNAC20-binding genomic fragments from maize leaves. Two independent biological replicates (IP_1 and IP_2) of DAP-seq libraries and their matched input controls were generated and subjected to high-throughput sequencing. The total clean reads and mapping rate for each replicate are listed in Supplemental Table S1. DAP-seq yielded three high-quality libraries: Input (negative control): 24.23 Gb clean reads, Q30 ≈ 93.6%, GC 44.3%. IP_1 (replicate 1): 27.58 Gb, Q30 ≈ 94.3%, GC 40.8%. IP_2 (replicate 2): 31.90 Gb, Q30 ≈ 93.4%, GC 40.8%. All datasets exceeded Q20 ≥ 97.6% with N content < 0.01%, meeting the criteria for downstream alignment and peak calling (Table S1). The mapping rates for the DAP-seq data were high for all samples, with over 97% of reads successfully aligned to the reference genome. The unique mapping rates were around 15%, indicating a good balance between multi-mapped and uniquely mapped reads (Table S2).

**Figure 1:**
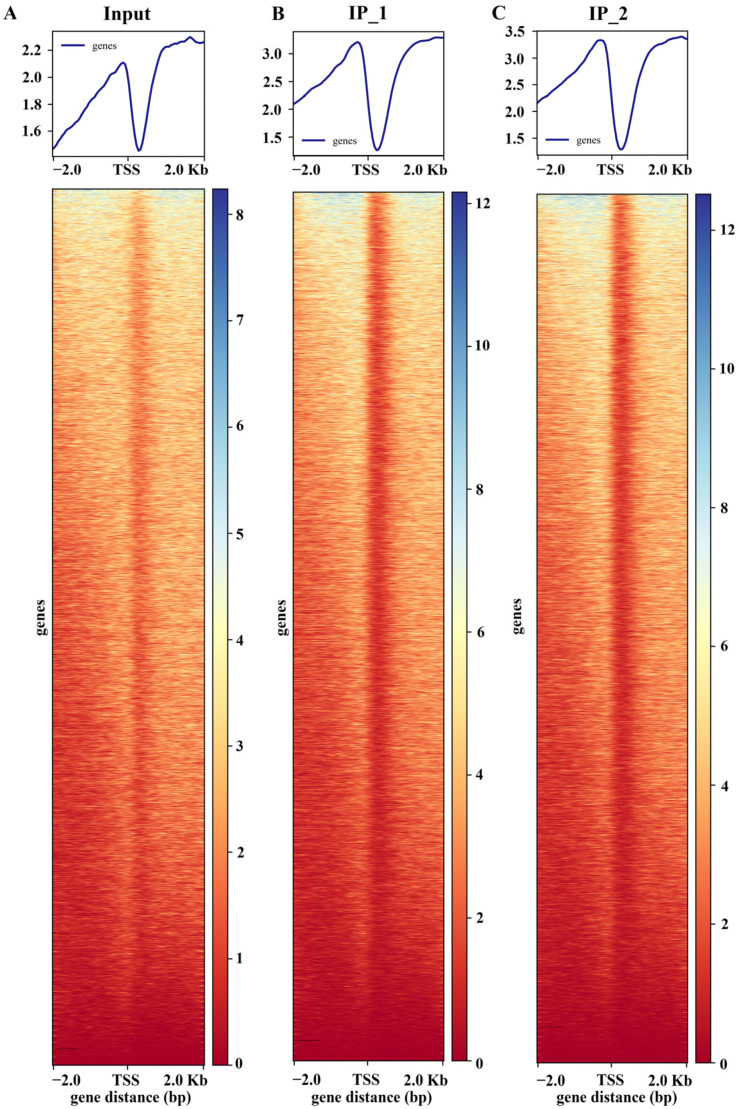
ZmNAC20 enrichment profile surrounding the transcription start site (TSS). Clean reads from IP_1, IP_2, and input were aligned to the maize reference genome, and plotted as average coverage per base pair across a 4 kb window (±2 kb) centered on the TSS of all annotated genes. The signal intensities of both input and IP samples were normalized to the same depth. The y-axis shows normalized read count (genes).

**Figure 2:**
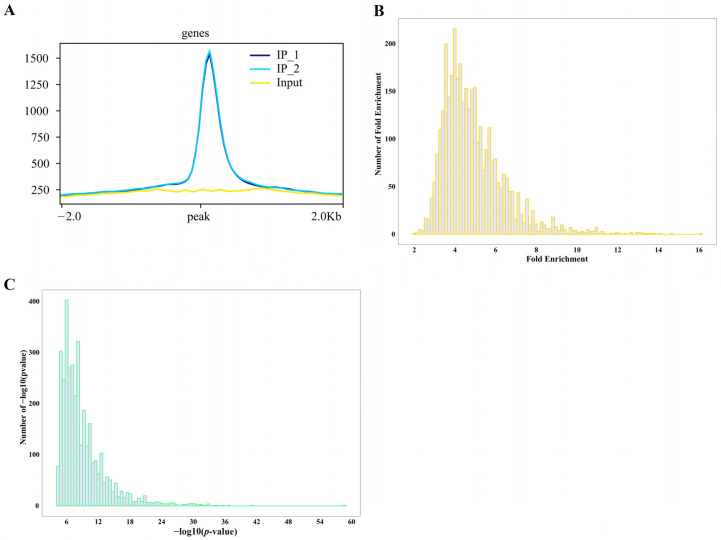
Distribution of ZmNAC20 IP peaks identified by DAP-seq. (**A**) Signal enrichment at peak centers. All identified peaks were centered and the average sequencing depth for both IP (IP_1 and IP_2) and input libraries was calculated in a ±2 kb window. (**B**) The number of peaks (*y*-axis) binned by their fold enrichment score (*x*-axis). Most peaks exhibit 8- to 12-fold enrichment over input, with the distribution tailing abruptly beyond 16-fold, indicating robust but moderate occupancy levels genome-wide. (**C**) Distribution of −log_10_ (*p*-value) per peak; the vast majority fall between 5 and 20.

Both DAP-seq replicates (IP_1 and IP_2) of maize ZmNAC20 displayed a sharp, highly overlapping peak within ±2 kb of the TSS. The summit is precisely positioned ~200 bp upstream of the TSS, with nearly identical signal intensities that are markedly above the input background (Figure 1). This indicates that ZmNAC20 binds in a highly specific and reproducible manner to the proximal promoter region at −200 bp, exhibiting a typical characteristic of transcription–initiation regulation and providing a robust basis for downstream peak calling, motif mining, and functional annotation of target genes. To visualize signal enrichment at the peak regions, we anchored all peaks at their centers and calculated the average read depth for both IP and input libraries across a ±2 kb window (Figure 2A). The resulting profile shows the metagene-like distribution of reads, illustrating the enrichment of the IP signal relative to input across the flanking 2 kb regions.

DAP-seq fold enrichment analysis revealed that ZmNAC20 binding sites are consistently enriched 8–12-fold relative to the input control. Very few peaks exceed 14-fold enrichment, and none reach the extreme values (>20-fold) often seen for highly amplified or repetitive regions (Figure 2B). This narrow, mid-range enrichment profile suggests that ZmNAC20 binds specifically, yet without strong amplification bias, providing target regions for downstream motif and functional analyses. DAP-seq peak calling yields exceptionally significant *p*-values for ZmNAC20-associated regions. Over 80% of all peaks exhibit −log10 (*p*-values) ≥ 36 (equivalent to *p* ≤ 10^−36^), far exceeding the conventional genome-wide threshold of 10^−8^ (Figure 2C). This left-skewed distribution confirms that the identified ZmNAC20 sites are highly reproducible and essentially free from a random background, providing a stringent and reliable set of direct target loci for subsequent functional validation.

### 2.2. Genome-Wide Profiling of ZmNAC20-Binding Motifs in Maize

**Figure 3:**
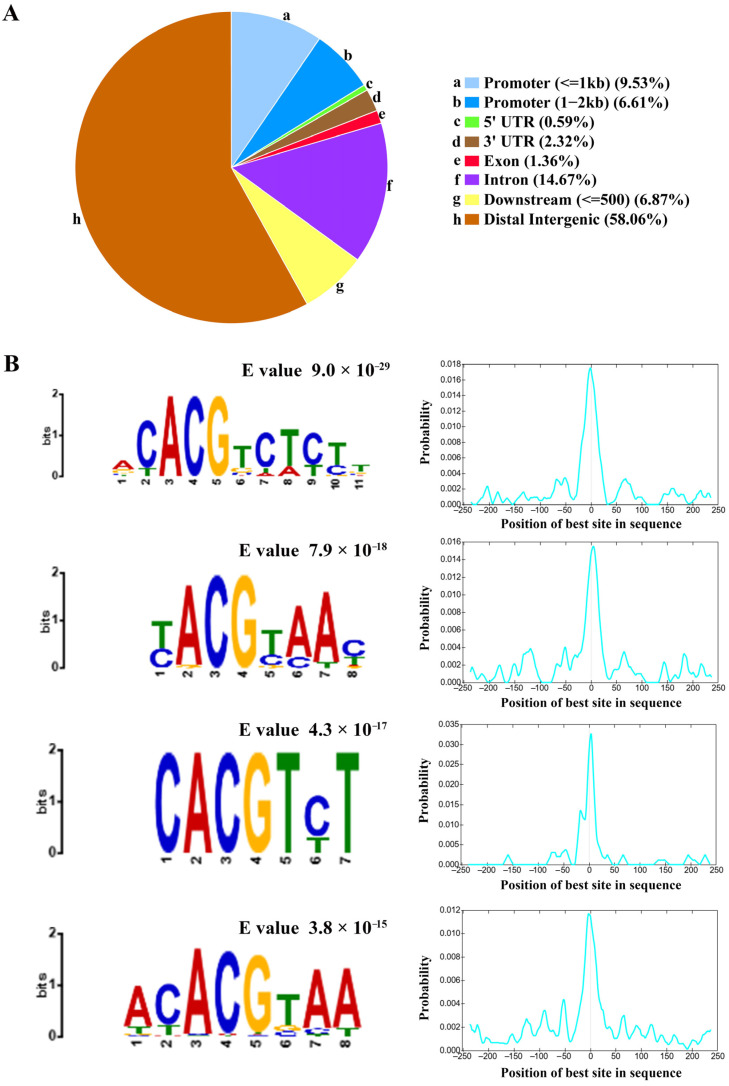
Genomic localization and de novo motif discovery of ZmNAC20 DAP-seq peaks. (**A**) The pie chart shows the percentage of peaks that overlap ten canonical genomic features: (a) promoter (≤1 kb upstream of the TSS), (b) promoter (1–2 kb), (c) 5′UTR, (d) 3′UTR, (e) exon, (f) intron, (g) downstream (≤500 bp), and (h) distal intergenic regions. Roughly 15% of all ZmNAC20 peaks reside within 2 kb of the TSS (promoter), while the remaining sites are distributed among gene bodies (5′UTR, exon, intron) and distal intergenic sequences, underscoring a primary but not exclusive role for ZmNAC20 in promoter-proximal regulation. (**B**) Top four de novo motifs recovered by MEME from the 200 bp sequences centered on peak summits, with their E values and positional distributions relative to the summit (0 bp). The most significant motif (E = 9.0 × 10^−29^) is the ACACGTCTCTT sequence; the second (E = 7.9 × 10^−18^) resembles the T(C)ACGT(C)A(C)AC sequence; the third (E = 4.3 × 10^−17^) is the CACGTC(T)T sequence; and the fourth (E = 3.8 × 10^−15^) is the ACACGTAA sequence. Each motif shows a sharp peak at the summit, confirming sequence-specific binding of ZmNAC20. Motif positional probability distribution (CentriMo output). *X*-axis: position of the best motif site in each peak sequence relative to the center (0 bp). *Y*-axis: probability density of occurrence at that position. Dashed line marks the sequence center (0 bp).

A consensus set of 3537 peaks reproducibly identified in both replicates was designated as the core ZmNAC20 binding regions (Table S3). The binding intervals spanned 240–1000 bp, and displayed a sharp peak centered at the transcription start site (TSS) (Figure 2A). Fine-scale annotation revealed that ZmNAC20 occupancy spans the genome: 58.06% of peaks reside in intergenic regions, 14.67% in introns, 6.87% within 500 bp downstream of genes, and only 1.36%, 0.59% and 2.32% in exons, 5′UTRs and 3′UTRs, respectively. A total of 16.14% of sites fall inside the 2 kb promoter regions, consistent with ZmNAC20 acting as a cis-regulatory TF that primarily modulates transcription initiation (Figure 3A). This distribution underscores ZmNAC20’s canonical role in transcriptional initiation, yet implies extensive long-range or enhancer-mediated gene regulation. ZmNAC20 peaks were evenly scattered along the entire length of all ten maize chromosomes, indicating a lack of chromosome-level preference.

To further elucidate the DNA-binding specificity of ZmNAC20, we performed de novo motif discovery on the binding regions defined by DAP-seq using the MEME Suite (meme-suite.org). De novo motif discovery revealed four highly enriched cis-elements (motif-1, motif-2, motif-3, motif-4) centered exactly at peak summits (Figure 3B, Table S4). The most abundant of these (549 peaks) conformed to the core sequence ACACGTCTCTT (E value, 9.0 × 10^−29^). We additionally identified three further motifs—T(C)ACGT(C)A(C)AC (E value, 7.9 × 10^−18^), CACGTC(T)T (E value, 4.3 × 10^−17^), and ACACGTAA (E value, 3.8 × 10^−15^)—present in 190, 163, and 232 peaks, respectively (Figure 3B, Table S4). CentriMo analysis shows that, within the 200 bp windows centered on DAP-seq peaks, the best-matching sites of the target motif are sharply concentrated at the sequence midpoint (0 bp) (Figure 3B). The significant enrichment of the motifs at the peak center was consistent with the canonical distribution expected for transcription factor binding sites. These results suggest that ZmNAC20 acts as a sequence-specific DNA-binding protein that recognizes both promoter and enhancer contexts.

### 2.3. ZmNAC20 Target Genes-Mediated Pathways

**Figure 4:**
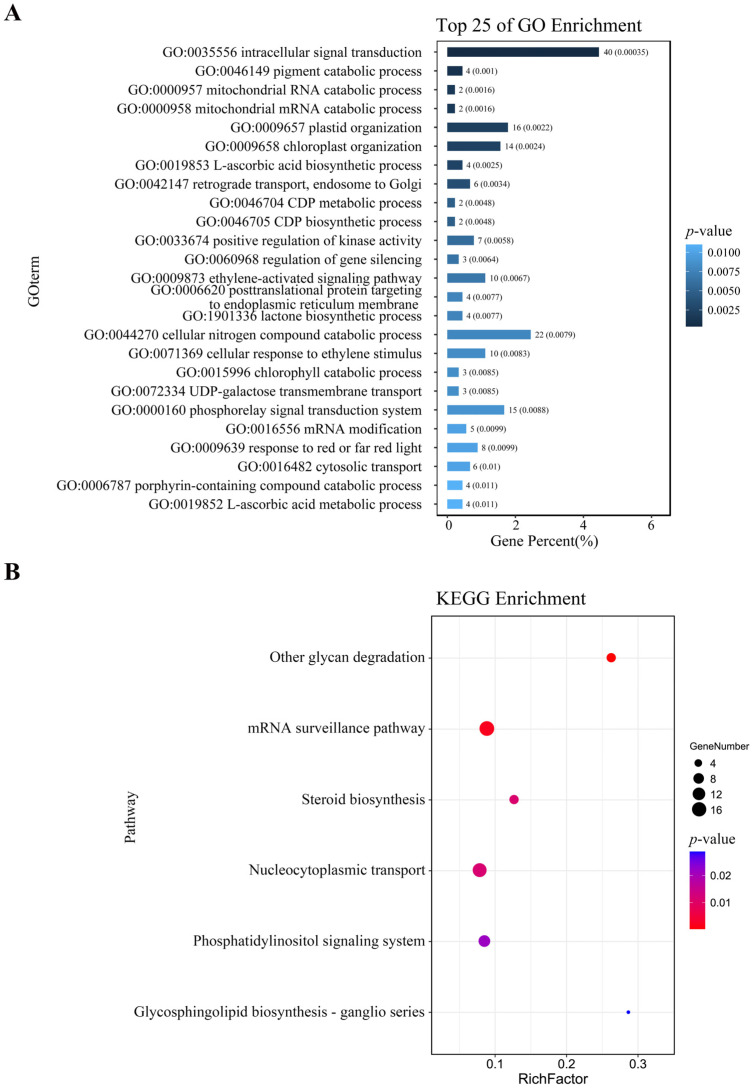
Functional landscape of ZmNAC20 target genes: GO biological processes and KEGG pathway enrichment. (**A**) Top 25 enriched Gene Ontology (biological process) terms among ZmNAC20 target genes. Bars indicate the percentage of input genes annotated to each term; numbers above bars give the exact gene count. *p*-values (Fisher’s exact test, unadjusted) are shown on the right. None of the terms remain significant after Benjamini–Hochberg correction (q-value ≥ 0.05); unadjusted values are retained only to illustrate relative ranking. Full *p*-values and q-values are provided in Table S8. (**B**) Top 6 enriched KEGG pathways among ZmNAC20 target genes. Bubble size reflects the number of input genes assigned to each pathway; RichFactor = (gene number in input)/(total genes in pathway). Color scale indicates unadjusted *p*-value (Fisher’s exact test). All pathways are non-significant after multiple-testing correction (q-value ≥ 0.05); see Table S9 for complete statistics.

After mapping the DAP-seq peaks to the genome, a total of 1484 genes were identified (Table S5). Further analysis revealed that 43 of these genes were redundant (Table S6), likely due to multiple peaks targeting the same gene or high sequence similarity among gene family members. For genes with multiple peaks, the one with the highest fold change value was retained (highlighted with red color, Table S6). After removing duplicates, 1439 unique target genes remained (Table S7). GO enrichment of the 1439 ZmNAC20-bound genes revealed a significant over-representation of 25 biological processes with the lowest raw *p*-values (Figure 4A; unadjusted Fisher’s exact test). The strongly enriched terms concern intracellular signal transduction, chloroplast organization, and the phosphorelay signal transduction system (Figure 4A). However, none of these terms remain significant after Benjamini–Hochberg correction (q ≥ 0.05; Table S8); they are therefore presented only to indicate relative biological relevance. These functions illustrate that the primary function of ZmNAC20 is to regulate signaling and organelle organization networks during plant responses. KEGG pathway enrichment of the 1439 ZmNAC20-bound genes uncovered six significantly over-represented pathways (*p* ≤ 0.05) (Figure 4B). As with the GO analysis, all adjusted q-values exceed 0.05 (Table S9), so the pathways should be viewed as exploratory rather than statistically significant. The largest functional cluster centered on steroid biosynthesis, nucleocytoplasmic transport, and the phosphatidylinositol signaling system (Figure 4B). These pathways illustrate that ZmNAC20 participates in steroid biosynthesis and signal transduction.

### 2.4. Drought-Responsive Targets of ZmNAC20 Revealed by Integrative DAP-Seq and RNA-Seq

**Figure 5:**
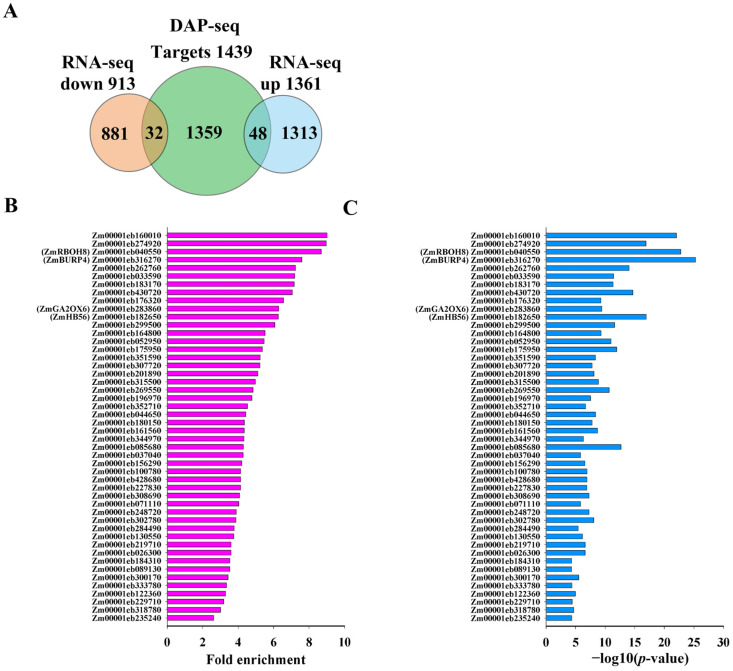
Integration of DAP-seq and RNA-seq identifies direct targets of ZmNAC20 under dehydration. (**A**) Venn diagram showing the overlap between 1439 ZmNAC20-bound genes (DAP-seq) and 2474 dehydration-responsive DEGs (RNA-seq). Bar graph summarizing the 80 direct targets: 48 up-regulated and 32 down-regulated in ZmNAC20-OE plants after 3 h of dehydration. (**B**) DAP-seq binding strength of ZmNAC20 on the 48 directly activated targets. The bar chart shows fold enrichment values for each gene. (**C**) Statistical significance of ZmNAC20 binding to the 48 directly activated targets. Bars represent −log_10_ (*p*-value) for each gene. Genes discussed in the text are labeled with their symbol names from maizeGDB database (**C**).

To uncover the genes that are directly controlled by ZmNAC20 under dehydration, we integrated DAP-seq and RNA-seq datasets. RNA-seq data were provided in our previous study [26]. RNA-seq was performed on three biological replicates each of wild-type B104 (WT-R1, WT-R2, WT-R3) and *ZmNAC20-OE* (OE-R1, OE-R2, OE-R3) maize. Detached leaves were subjected to a 3 h dehydration treatment before total RNA extraction, enabling a direct comparison of the transcriptomes between the wild-type B104 and *ZmNAC20-OE* under identical water-deficit stress. DAP-seq identified 1439 genomic loci bound by ZmNAC20 in vitro (Table S7). After a 3 h dehydration treatment, RNA-seq of ZmNAC20-over-expressing (OE) transgenic plants and wild-type B104 revealed 2474 differentially expressed genes (DEGs), of which 1361 were up-regulated and 913 were down-regulated [26]. Cross-referencing the two datasets yielded 80 genes that are both bound by ZmNAC20 and transcriptionally regulated by it (Figure 5A, Tables S10 and S11). Among these direct targets, 48 were induced and 32 were repressed in the OE lines (Tables S12 and S13), indicating that ZmNAC20 functions as both an activator and a repressor in the dehydration response network.

To quantify the in vivo affinity of ZmNAC20 for its activated targets, we plotted the DAP-seq fold enrichment values for the 48 genes that were both bound in the DAP-seq assay and significantly up-regulated in ZmNAC20-over-expressing plants after 3 h of dehydration. The enrichments ranged from ~2- to 10-fold (median ≈ 4.5), indicating robust and variable occupancy across promoters (Figure 5B). Several well-characterized stress-related loci, such as respiratory burst oxidase 8 (*ZmRBOH8*, *Zm00001eb040550*), BURP domain-containing protein-RD22-like 4 (*ZmBURP4*, *Zm00001eb316270*), gibberellin 2-oxidase 6 (*ZmGA2ox6*, *Zm00001eb283860*), and homeobox-transcription factor 56 (*ZmHB56*, *Zm00001eb182650*) displayed above-average binding strength (>6-fold), supporting their classification as direct targets of ZmNAC20. The above gene symbols refer to the maizeGDB database (www.maizegdb.org, accessed on 3 December 2025). To evaluate the statistical significance of ZmNAC20 binding to its 48 directly activated targets, we extracted the −log_10_ (*p*-value) for each peak from the DAP-seq dataset. All peaks surpassed the genome-wide significance threshold (−log_10_ (*p*-value) > 2), with the majority exceeding 10 and several stress-related loci—e.g., *ZmRBOH8*, *ZmBURP4*, *ZmGA2ox6*, and *ZmHB56*—reaching values above 20 (Figure 5C). These high −log_10_ (*p*-values) confirm that the observed enrichments are highly unlikely to arise by chance, reinforcing the classification of these 48 genes as direct targets of ZmNAC20.

One motif ACACGTTTCCC (classified as motif 1) was identified in the fourth intron of *ZmRBOH8* (*Zm00001eb040550_T001*), located on the antisense strand at 2464 bp downstream of the start codon. Two motifs CACGTATA (classified as motif 2) and ACACGCCA (classified as motif 4) were identified in the promoter of *ZmBURP4* (*Zm00001eb316270_T001*), located on the sense strand at 989 bp and 1325 bp upstream of the start codon, respectively. Two motifs CACGCCAC (classified as motif 2) were identified in the promoter of *ZmGA2OX6* (Zm00001eb283860_*T001*), located on the sense strand at 578 bp and 684 bp upstream of the start codon, respectively. A motif CACGCCTG (classified as motif 2) was identified in the first exon of *ZmHB56* (*Zm00001eb182650_T001*), located on the sense strand at 54 bp upstream of the start codon.

**Figure 6:**
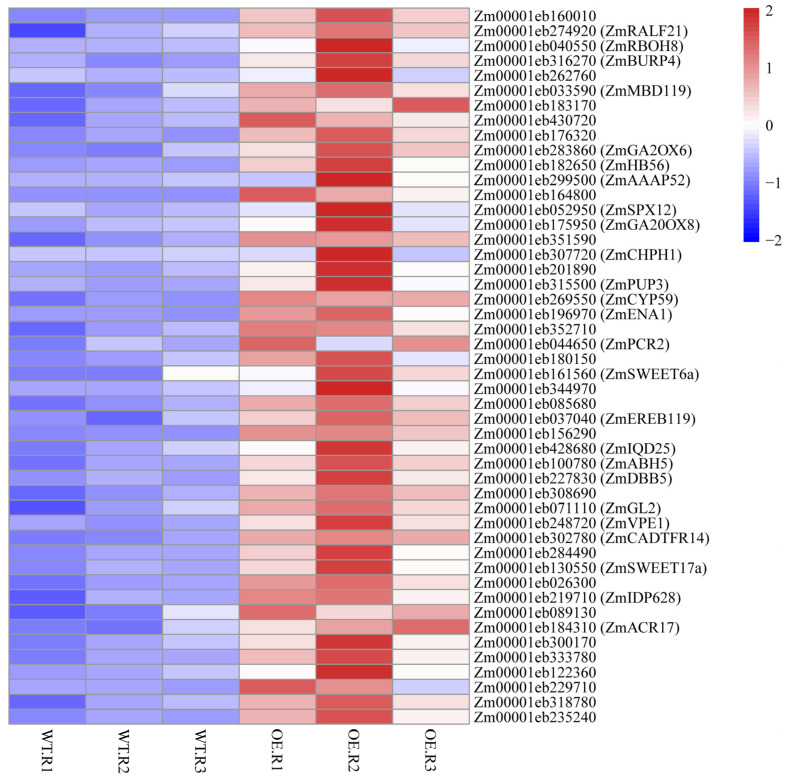
Heat-map of RNA-seq log_2_-fold changes for the 48 directly activated ZmNAC20 targets. Each row represents one gene; color scale (right) ranges from blue (repressed) to red (induced). Genes discussed in the text are labeled with their symbol names from maizeGDB database (accessed on 31 December 2025). Genes lacking the symbol in MaizeGDB are shown without annotation because no curated description is currently available.

To visualize the transcriptional response of the 48 ZmNAC20-activated targets, we generated a heat-map of their RNA-seq log_2_-fold changes in transgenic plants over-expressing ZmNAC20 (OE) versus B104 control plant after 3 h of dehydration (Figure 6). The majority of genes exhibited strong induction (red), whereas a small subset remained nearly unchanged or weakly repressed (blue), confirming that ZmNAC20 up-regulates this target set. Notably, well-characterized stress regulators such as *ZmRBOH8*, *ZmBURP4*, *ZmGA2ox6*, and *ZmHB56* clustered among the most highly up-regulated genes, underscoring their central role in the ZmNAC20-mediated dehydration response.

## 3. Discussion

NAC transcription factors are key players in coping with dynamic abiotic stresses [43]. ZmNAC20 is a positive regulator of maize drought tolerance [26], yet the genome-wide circuitry through which it exerts this function had remained largely unknown. By coupling high-resolution DAP-seq with complementary RNA-seq we have now charted a comprehensive, experimentally validated regulon that encompasses 1439 unique, candidate targets (Table S7). The data reveal ZmNAC20-mediated pathways that provide a mechanistic explanation for its previously reported physiological activity (Figure 4): a bifunctional regulator that can both activate and repress transcription. Integration with dehydration-responsive RNA-seq showed that 48 targets are significantly up-regulated while 32 are down-regulated in *ZmNAC20-OE* lines. The activator function is dominant in the drought response, but the repressive component involves context-dependent recruitment of co-repressors or chromatin modifiers. Future chromatin immunoprecipitation–mass spectrometry (ChIP-MS) experiments will be necessary to identify the protein partners that switch ZmNAC20 from an activator to a repressor mode. Across plants, some NACs behave primarily as transcriptional activators (e.g., ZmNST3) [36], some NACs act as dedicated repressors (e.g., ZmNAC49) [35], and several NACs can switch between activation and repression depending on promoter context or interacting partners (e.g., ANTHER INDEHISCENCE FACTOR (AIF), ARABIDOPSIS THALIANA ACTIVATION FACTOR 2 (ATAF2)) [44,45].

The ZmNAC20 profile showing mostly binding in distal intergenic regions is an expected limitation of the in vitro DAP-seq assay. In the test tube the transcription factor binds naked DNA in the absence of native cofactors and chromatin context; because the entire genome is equally accessible, the protein can associate with sequences that would be occluded or poorly accessible in vivo. Consequently, a sizeable fraction of peaks falls outside promoter regions and appear in the much larger intergenic space, reflecting the technical nature of the method rather than promiscuous binding in plants. Consistent with this view, DAP-seq surveys of the maize NAC transcription factor ZmNST3 revealed that 64.34% its binding peaks reside in intergenic regions [36]. Whether these distal sites function in vivo will require locus-specific ChIP-seq perturbation.

Based on the function of their homologous genes, and through DAP-seq and RNA-seq analyses, *ZmRBOH8*, *ZmBURP4*, *ZmHB56*, and *ZmGA2OX6* were identified as candidate target genes mediating the drought stress response regulated by ZmNAC20 (Figure 5). ZmRBOH8 includes an ROS-generating NADPH oxidase domain; however, its precise role in maize drought tolerance remains to be functionally validated. ZmBURP4 belongs to the BURP domain-containing family (named after BNM2, USP, RD22, and polygalacturonase isozyme). Over-expression of *SlRD1*—a BURP member in *Solanum lycopersicum*—has been shown to confer enhanced drought tolerance [46]. ZmHB56, as an HD-ZIP protein (homeodomain–leucine zipper protein), belongs to a large family of plant-specific transcription factors which are involved in regulating diverse development processes and stress responses. ZmHB56 has been shown to regulate seed size. Over-expression of *ZmHB56* in Arabidopsis and maize increased seed size [47]. ZmHDZ9, the homologous gene of ZmHB56, contributes to drought resistance improvement by modulating ABA and lignin accumulation [48].

ZmGA2OX6 is an enzyme that regulates gibberellin (GA) degradation. The balance of GA metabolism is essential for plant growth, development, and stress adaptation. By regulating GA biosynthesis, catabolism, and signal transduction, plants coordinate growth with environmental responses, thereby maintaining physiological function and survival under fluctuating conditions [49,50,51]. GA levels are modulated by various environmental cues, primarily through the regulation of three small gene families acting in the late stages of GA metabolism. One of them is GA2-oxidases (GA2ox), which inactivate bioactive GAs or their C-19 and C-20 precursors [52,53]. Among the numerous GAs, most are either precursors or inactive metabolites of the bioactive forms; only a few, such as GA1, GA3, GA4, and GA7, possess biological activity [54]. Under drought stress, GA levels dynamically shift to help plants cope with water deficit. In rice, most GA metabolism-related genes are regulated by water deficiency, leading to reduced GA content and consequent growth inhibition [55]. In tomatoes, drought stress enhances the expression of GA catabolic genes, lowering GA levels, which in turn promotes stomatal closure and improves drought tolerance [53]. Arabidopsis AtGAMT1 encodes a methyltransferase that catalyzes the methylation of bioactive GAs, yielding inactive GA methyl esters. Ectopic expression of AtGAMT1 in tomatoes reduces GA content and significantly enhances drought resistance [56]. These findings suggest that reducing GA levels or suppressing GA signaling can contribute to improved drought tolerance in plants.

Our study was conducted in juvenile leaves under controlled dehydration; field drought entails additional layers such as high irradiance, nutrient fluctuation, and pathogen pressure that may reshape the ZmNAC20 landscape. Single-cell or spatially resolved ChIP-seq will be required to determine whether the observed binding pattern is uniform across cell types or enriched in specific tissues, such as guard cells and bundle-sheath. Moreover, the current motif catalog is based on 3537 peaks; expanding the analysis to weaker yet reproducible peaks may uncover auxiliary motifs that fine-tune binding affinity. Genome editing of the motifs in native promoters followed by drought phenotyping will provide causal evidence that these sites are necessary for ZmNAC20-dependent activation.

## 4. Materials and Methods

### 4.1. DAP-Seq Assay

Plant materials and growth conditions were slightly modified based on the previous study [57]. Seeds of maize (*Zea mays* L.) inbred line B104 were surface-sterilized, sown in pots, and grown under greenhouse conditions (28 °C; 16 h light/8 h dark) with regular irrigation. DAP-seq assay was conducted according to the method described in the previous study [58]. Leaves of 12-day-old seedlings were harvested, immediately frozen in liquid nitrogen, and used for genomic DNA (gDNA) library construction. The ZmNAC20 coding sequence was cloned into pFN19K HaloTag T7 SP6 Flexi vector and expressed with the TNT SP6 High-Yield Wheat Germ Protein Expression System (Promega, Madison, WI, USA). Recombinant protein was immobilized on Magne HaloTag Beads (Promega, Madison, WI, USA) and incubated with adapter-ligated gDNA libraries. After washing, bound DNA was eluted and sequenced on an Illumina NovaSeq 6000 in two technical replicates. Beads without protein served as input negative controls. After extraction, DAP-enriched DNA was sheared to 100–300 bp fragments by ultrasonication. Following end-repair, 3′-A tailing and ligation of Illumina adapters, size-selected fragments were PCR-amplified to generate the final sequencing library. Libraries were quantified, quality-controlled and sequenced on an Illumina NovaSeq™ 6000 platform by Gene Denovo Biotechnology Co. (Guangzhou, China).

### 4.2. DAP-Seq Data Processing and Peak Annotation

Clean reads were aligned to the reference genome (Zm-B73-REFERENCE-NAM-5.0) with Bowtie 2 (version, 2.2.5) [59]. Read coverage across the transcription start site (TSS)–transcription end site (TES) body and 2 kb flanking regions was quantified using DeepTools (version, 3.2.0) [60]. Peaks were called with MACS2 (version, 2.1.2) using the corresponding input library as the control. Peaks were called with MACS2 using the paired-end mode, keeping only sites with a q-value < 0.05. Read depth profiles were generated in normalized signal-per-million-reads (SPMR) units for downstream visualization [61]. Peak-associated genes were annotated with ChIPseeker (version, 1.16.1); genomic distributions (intergenic, upstream, downstream, exon, intron) were simultaneously calculated [62]. All sequence reads have been deposited in the NCBI Sequence Read Archive (SRA) under project PRJNA1365078.

### 4.3. GO and KEGG Enrichment Analysis

Gene Ontology (GO) and Kyoto Encyclopedia of Genes and Genomes (KEGG) enrichment were analyzed by the OmicShare tool (https://www.omicshare.com, accessed on 3 December 2025). GO terms with adjusted *p* ≤ 0.05 were considered significantly enriched, revealing the principal biological functions of the target genes. KEGG pathway enrichment was performed with the same hypergeometric test and FDR correction (FDR ≤ 0.05) to identify significantly over-represented metabolic and signal–transduction pathways among the peak-associated genes.

### 4.4. Motif Analysis

Conserved DNA binding motifs were identified with the MEME suite (http://meme-suite.org, accessed on 3 December 2025). MEME (http://meme-suite.org/tools/meme, accessed on 3 December 2025) and DREME (http://meme-suite.org/tools/dreme, accessed on 3 December 2025) were employed to detect long (8–15 bp) and short consensus sequences (3–8 bp), respectively.

### 4.5. Dehydration Treatment

Above-ground tissues of three-true-leaf B104 and ZmNAC20-over-expressing transgenic plants (*ZmNAC20-OE*) of maize seedlings were excised, placed in a plant growth chamber (28 °C, 30% relative humidity, 16,800 lux), and dehydrated for 3 h.

### 4.6. RNA-Seq Data Analysis

RNA-seq data were obtained from our previous study [26]. Three biological replicates of wild-type B104 (WT-R1, WT-R2, WT-R3) and ZmNAC20-OE (OE-R1, OE-R2, OE-R3) were analyzed. Total RNA was isolated from detached leaves dehydrated for 3 h. Detailed analytical procedures are described in the original publication [26]. All sequence reads have been deposited in the NCBI Sequence Read Archive (SRA) under project PRJNA893876.

## 5. Conclusions

This work identified the direct ZmNAC20-regulated target genes at genome scale using DAP-seq. Four significantly enriched DNA motifs were recovered, underpinning 1439 ZmNAC20-bound genes. Functional enrichment and integrative analyses reveal that ZmNAC20 orchestrates a multi-layered network that intersects signal transduction, chloroplast retrograde signals and metabolic adaptation. Integration of DAP-seq with RNA-seq distilled a stringent set of 80 direct targets (48 activated, 32 repressed) by ZmNAC20. The 48 directly activated genes, including *ZmRBOH8*, *ZmBURP4*, *ZmGA2OX6* and *ZmHB56*, form a candidate module that translates ZmNAC20 binding into rapid physiological adjustments. These findings provide a comprehensive molecular framework for understanding how a single NAC transcription factor can confer enhanced drought tolerance and offer a prioritized gene list for precision breeding or genome editing in maize and related cereals.

## Data Availability

The original contributions presented in this study are included in the article/Supplementary Material. Further inquiries can be directed to the corresponding authors.

## Supplementary Materials

The following supporting information can be downloaded at: https://www.mdpi.com/article/10.3390/plants15030423/s1, Table S1: Total number of clean reads by DAP-seq, Table S2: The mapping rate for each replicate of DAP-seq, Table S3: List of DAP-seq enriched peaks, Table S4: List of ZmNAC20-binding motifs, Table S5: List of ZmNAC20-regulated direct target genes, Table S6: List of 43 redundant ZmNAC20 target genes, Table S7: List of ZmNAC20-regulated direct unique target genes, Table S8: GO terms of ZmNAC20-regulated direct unique target genes, Table S9: KEGG pathways of ZmNAC20-regulated direct unique target genes, Table S10: List of 48 up-regulated genes by DAP-seq and RNA-seq, Table S11: List of down-regulated genes by DAP-seq and RNA-seq, Table S12: List of 48 up-regulated genes by ZmNAC20 using RNA-seq, Table S13: List of 33 down-regulated genes by ZmNAC20 using RNA-seq.
